# CT Anatomical Features and Dimensions of the Rabbit Adrenal Glands

**DOI:** 10.3390/vetsci12070632

**Published:** 2025-07-02

**Authors:** Kamelia Stamatova-Yovcheva, Rosen Dimitrov, Diyana Vladova, David Yovchev, Hristo Hristov, Vladi Nedev, Nikolay Goranov, Avche Dineva

**Affiliations:** 1 Department of Veterinary Anatomy, Histology and Embryology, Faculty of Veterinary Medicine, Trakia University, 6000 Stara Zagora, Bulgaria; rdimitrov288@gmail.com (R.D.); diana_vladova@abv.bg (D.V.); vetjovchev@yahoo.com (D.Y.); klokkende@abv.bg (H.H.);; 2 Department of Veterinary Surgery, Faculty of Veterinary Medicine, Trakia University, 6000 Stara Zagora, Bulgaria; nedevladi@gmail.com (V.N.); nikolay.goranov@trakia-uni.bg (N.G.)

**Keywords:** CT, rabbit, adrenal glands

## Abstract

**Background:** Adrenal glands are situated cranially and in close proximity to both kidneys. The rabbit is a preferred animal pet species and is also used as an experimental model in research. The aim of this study was to investigate the CT anatomical features of the rabbit adrenal glands, using the CT device SOMATOM. **Methods**: Ten sexually mature clinically healthy rabbits, with body weights from 2.5 to 3.0 kg were used. They were separated into two groups—five males and five females. CT algorithm: Transverse, sagittal, and dorsal CT studies were performed. The animals were positioned in supine recumbency. They were anesthetized with 15 mg/kg Zoletil^®^ 50. Helical CT was carried out. The body was scanned from the intrathoracic part of the abdominal cavity to the pelvic inlet. CCD, DVD, and MLD dimensions were measured. **Results:** Transverse CT scans at the Th12 and L1 level visualized the topography of the right and left adrenal glands. Sagittal CT scans of *Regio abdominis*, 10 mm to the right and to the left of the median plane, showed the right and left adrenal glands as findings with intermediate attenuation. The dorsal CT investigation of *Regio abdominis* 30 mm and 45 mm ventral to the spine demonstrated both glands in the vicinity of abdominal organs and large blood vessels. CCD of the right adrenal gland was 6.9 ± 0.5 mm, DVD of the same gland was 5.7 ± 0.7 mm, and MLD was—3.9 ± 0.3 mm. CCD of the left adrenal gland was 5.4 ± 0.6 mm, DVD was—4.0 ± 1.1 mm, and MLD was—4.1 ± 0.9 mm. **Conclusions**: CCD and DVD of the right adrenal gland were greater than the same parameters of the left gland. MLD of the right adrenal gland was lower than the same parameter measured for the left one.

## Simple Summary

The CT is a contemporary and modern imaging method to study the rabbit adrenal glands. The right gland was localized on the right of the median plane, in the dorsal abdominal quarter. It was detected dorsally and in close proximity to the contrast-enhancing *v. cava caudalis*, the *aorta abdominalis,* and the liver. The soft tissue attenuation of the *gl. adrenalis dextra* was relatively lower than that of the right liver lobe. The left gland was visible at a more caudal level of scanning compared to the right adrenal. It was at a distance from the left kidney. The obtained CT results may be applied in the quest for an appropriate approach for the treatment of rabbit-specific adrenal diseases.

## 1. Introduction

Adrenal glands (*gll. adrenales*) are paired endocrine organs, located in close proximity and cranially to both kidneys. They are dorsoventrally flattened and craniocaudally elongated. The adrenal glands are retroperitoneal organs, located medially to craniomedially to the right and left kidneys. Their topography resembles that of kidneys, as the right gland is more cranially located compared to the left. In general, the location of both glands is confined by the well-defined capsula adiposa of the respective kidney. The right and left adrenal glands are anatomically connected to the kidneys due to the presence of a well-defined adipose tissue between blood vessels and nerves. The medial border of the right adrenal gland is close to the *v. cava caudalis*, whereas the *margo medialis* of the left adrenal gland borders the abdominal aorta and *a. mesenterica cranialis*. The dorsal surface of the adrenal glands is less variable than the ventral one and is largely defined by the renal and vascular topography, while the ventral surface contacts the parietal peritoneum [1,2].

The adrenal glands of rabbits have an elliptical, elongated shape and are located cranially to the *extremitas cranialis* of the respective kidney and are separated from the kidney by adjacent blood vessels. The left gland is situated further away from the left kidney, unlike the right gland, which is anatomically closer in relation to the right kidney [1].

During recent years, New Zealand White rabbits have been used as experimental models in veterinary medical research, and for the study of the morphological and physiological features of adrenals in other animal species and humans. This animal species has become increasingly popular as a pet [3,4]. At the same time, rabbit meat production is also important worldwide [5].

As a biological species, the rabbit is susceptible to diseases of the adrenal glands, such as congenital adrenal hyperplasia, hyperadrenocorticism, neoplastic growths, adrenomegaly, etc. [6,7,8].

It is reported that the adrenals of the New Zealand rabbit are located cranially to the respective kidney [4]. Macromeasurements demonstrate that the right adrenal gland in the rabbit is longer than the left one. The thickness of both glands is similar. The width of the left gland exceeds that of the right one. The dimensions of the glands are not associated with the sex of animals [4].

In a post-mortem morphometry study on rabbit adrenals [9], the length, width, and height of the left adrenal gland were greater than the same measures of the right gland, irrespective of the sex.

Unlike lagomorphs, including rabbits, the adrenal glands of rodents have been studied. The macrometric parameters of adrenal glands in rodents are species-specific. In porcupines (*Hystrix cristata*), the left adrenal gland is wider than the right one [10]. *Galea spixii* demonstrates the opposite trend, e.g., the right adrenal of males and females is wider and longer than the respective parameters of the left gland [11]. The width of the adrenal glands is reported to be greater in female lowland pacas than in males [12].

The helical post-contrast CT is a non-invasive imaging technique for obtaining objective anatomical data about a variety of soft tissue structures and bones in small animals and pets. The obtained data are detailed and clinically relevant. CT imaging of the abdomen is a precise method for visualization of the liver, the spleen, the pancreas, the digestive organs, the adrenals, and the kidneys [13].

Dorsal CT scans of the rabbit abdomen at a distance of 20 mm from the spine allow observing only the left adrenal gland, the adjacent left kidney, and small intestinal loops. The gland shape is oval, and the attenuation on the gray scale is intermediate [14].

Computed tomography (CT) is an appropriate method for obtaining reliable imaging macrometric parameters of canine adrenals. The variations in glandular dimensions and shape are due to the levels of CT scans, and in some cases, to individual differences. In some CT scans, both glands are presented, and in others, only one of them is [15].

The CT study is described as a sufficiently definitive method for the visualization of canine adrenal glands and an appropriate approach for clinical studies. The variable soft tissue density characteristics obtained from adrenal gland imaging are used for differentiation of normal soft tissue findings from pathological adrenals [16].

CT is a relevant imaging method for the macromorphometric measurement of feline adrenal glands. The obtained results are consistent and correspond to the real values of the studied macroparameters. The length and height of the right and left adrenal glands have very close values, while the width of the right adrenal is greater than that of the left one [17].

The transverse post-contrast CT study of chinchilla’s adrenal glands provides information about the topography of both glands as retroperitoneal abdominal organs. At the L3 level, both adrenals are simultaneously visible. The right adrenal gland is presented as a hypoattenuated, ellipse-shaped soft tissue finding in close proximity to the *v. cava caudalis*. The density of the left gland is similar to that of the spleen, and its shape is oval. The technique turned out to be appropriate for obtaining real intravital data on the macroparameters of the studied organs. The height and the length of the right adrenal are greater than those of the left one. At the same time, the left adrenal width exceeds that of the contralateral gland [18].

Ultrasound (US) was applied as a method to study the US measurements of the rabbit adrenals. The size of the studied glands is related to the sex, weight, and diameter of the abdominal aorta [19].

The presented literature data demonstrate that data on the CT anatomical features and parameters of adrenal glands in the rabbit are insufficient. This was a sound reason to undertake the present study, which is aimed at obtaining reliable information on the subject to complete modern anatomical literature data and serve as a background model in clinical science and practice.

## 2. Materials and Methods

### 2.1. Materials

Ten clinically healthy sexually mature New Zealand White rabbits, 7 months of age, with a body weight of 2.5–3.0 kg were studied. The experimental animals were distributed in two groups—5 males and 5 females. The rabbits were from a specialized farm, belonging to the Agricultural Institute, Stara Zagora, Bulgaria

### 2.2. CT Protocol

This study was performed with the helical computed tomograph SOMATOM go.No, SN 168190, Simens Healthineers Headquarters, Forchheim Germany. The animals were positioned in supine recumbency. They were anesthetized with 15 mg/kg Zoletil^®^ 50 IM (tiletamine hydrochloride 125 mg and zolazepam hydrochloride 125 mg in 5 mL of solution) (Virbac, Carros-Cedex, France). The investigation was carried out on 29 May 2024, in the University Veterinary Hospital, Stara Zagora, Bulgaria.

The body was scanned from the intrathoracic part of the abdominal cavity to the pelvic inlet. Slice thickness was 0.7 mm, electric current intensity—30 mA, scanning time—from 0.8 s to 1.0 s, rotation time—up to 0.5 s6, rotational speed—360° in 0.8, 1, 2, 3, and 4 s; pitch—6, anode tension 130 kV, zoom 145%, WL 50 and WW 350, exposure time 1981 s, CTDIvol 1.67 mGy, converting filter—standard, tilt 0.5, level 35, FOV 50. The images were processed by DICOM. Transverse, sagittal, and dorsal slices were obtained. Transverse abdominal sections were obtained between the planes from the 11th thoracic to the 7th lumbar vertebrae. The sagittal and dorsal sections were reconstructed from a standard transverse view. For better anatomical orientation, the CT transverse images described as the position of the findings coincided with the position of the researcher. The results were stored in a DICOM format.

Transversal CT scans measured the dorsoventral dimension (corresponding to the thickness)—DVD—and the mediolateral dimension (corresponding to the width)—MLD—. Sagittal CT scans measured the craniocaudal dimension (corresponding to the length)—CCD—. The results were in mm and were interpreted using descriptive analysis in Statistica 8—StatSoftDELL (version 13.3.721).

The research arises from project 06/23 “Morphological and Imaging Anatomical Features of the White New Zealand rabbit adrenal glands”, Trakia University, Faculty of Veterinary Medicine, with leader Associate Professor Kamelia Stamatova-Yovcheva, Ph.D.

This study has been evaluated by permission No. 377, issued by the Ethics Committee of the Ministry of Agriculture and Food, Bulgarian Animal Safety Agency, with opinion No. 293 of 29 February 2024, Sofia, Bulgaria, in compliance the provisions of the Animal Protection Act in Bulgaria (promulgated in State Gazette No 13/8 February 2008) and the European Convention for the Protection of Vertebrate Animals Used for Experimental and Other Scientific Purposes (ETS No 123, OJ L 222, 24 August 1999, pp. 31–37). All animal experiments comply with the ARRIVE guidelines and were conducted in accordance with the U.K. Animals (Scientific Procedures) Act, 1986, and associated guidelines, EU Directive 2010/63/EU for animal experiments.

## 3. Results

### 3.1. Transverse Post-Contrast CT Anatomical Study

**Figure 1:**
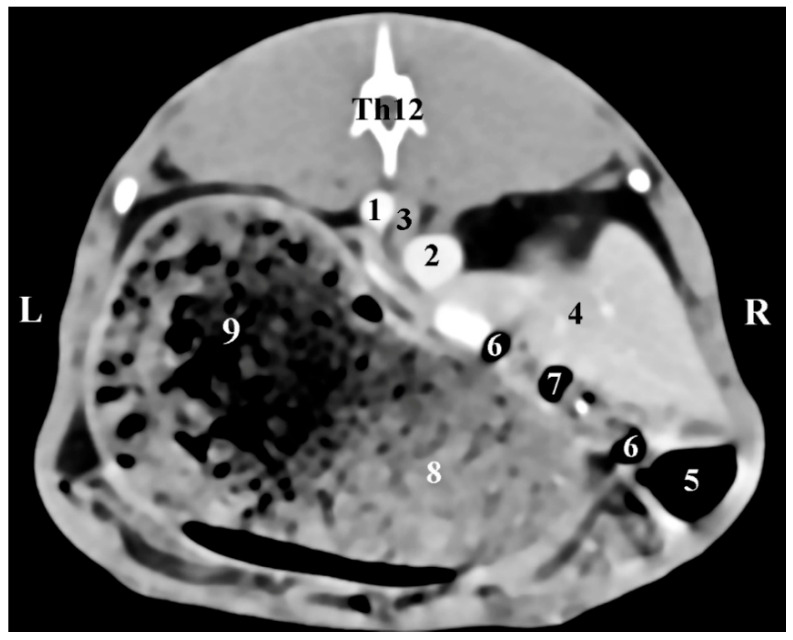
Transverse CT post-contrast anatomical study of the rabbit abdomen at the level of Th12. L—left; R—right. (1) *aorta abdominalis*; (2) *v. cava caudalis*; (3) right adrenal gland; (4) *lobus hepatis dexter*; (5) *caecum*; (6) *duodenum*; (7) *ileum*; (8) *corpus ventriculi*; (9) *fundus ventriculi*.

The transverse post-contrast CT anatomical investigation at the level of the 12th thoracic vertebra (Th12) gave complete information about the topographic location and shape of the right adrenal gland. The latter was a retroperitoneal soft tissue finding located in the intrathoracic part of the abdominal cavity. The gland was localized on the right of the median plane, in the dorsal abdominal quarter. It was detected dorsally and in close proximity to the contrast-enhancing *v. cava caudalis*, the *aorta abdominalis,* and the liver. The borders of the right adrenal were sharply delineated, which defined it as an ellipse-shaped structure. The soft tissue attenuation of the *gl. adrenalis dextra* was relatively lower than that of the right liver lobe (Figure 1).

**Figure 2:**
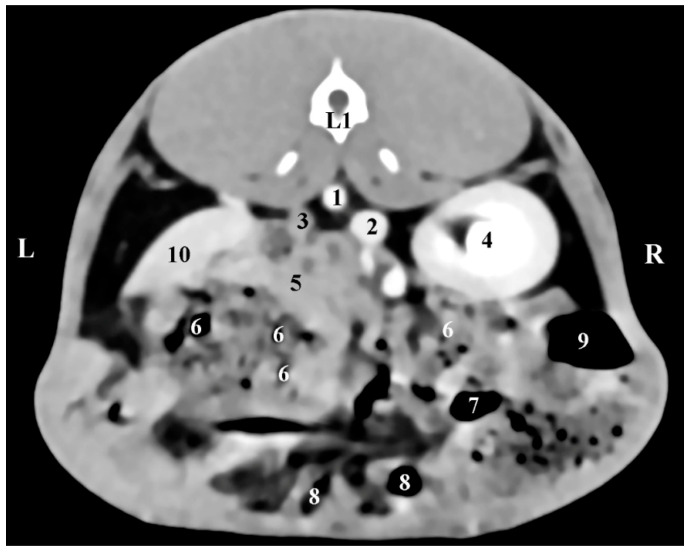
Transverse CT post-contrast anatomical study of the rabbit abdomen at the level of L1. L—left; R—right. (1) *aorta abdominalis*; (2) *v. cava caudalis*; (3) left adrenal gland; (4) right kidney; (5) small intestine; (6) *mesenterium*; (7) *colon descendens*; (8) *colon ascendens*; (9) *caecum*; (10) *lien*.

The left adrenal gland was visualized at the level of the 1st lumbar vertebra (L1). It was left to the median plane and in the dorsal abdominal cavity quarter. The gland was visible at a more caudal level of scanning compared to the right adrenal. *gl. adrenalis sinistra* had oval contours and was observed ventrally to the contrast-enhancing abdominal aorta and distant and craniomedially to the left kidney. The left adrenal density was similar to that of the ventrally located mesentery (Figure 2).

### 3.2. Sagittal Post-Contrast CT Anatomical Study

**Figure 3:**
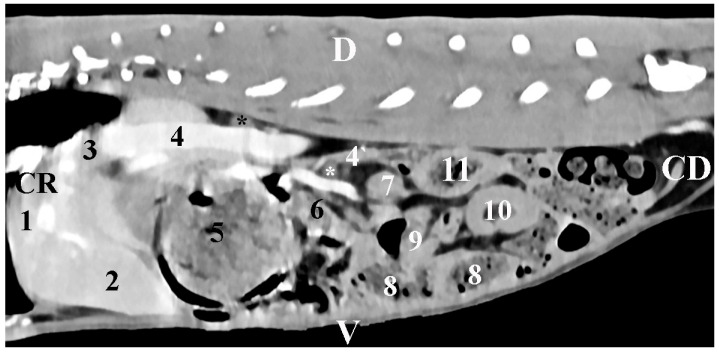
Sagittal CT post-contrast anatomical study of the rabbit abdomen at the level of the plane at 10 mm to the right of the spine. CR—cranial; CD—caudal; D—dorsal; V—ventral. (Black star) right adrenal gland; (white star) mesenterial fold; (1) *lobus hepatis dexter*; (2) *lobus hepatis sinister medialis*; (3) *lobus caudatus*; (4) *v. cava caudalis*; (4`) v. mesenterica cranialis; (5) *corpus ventriculi*; (6) *pars descendens duodeni*; (7) right kidney; (8) caecum; (9) jejunum; (10) *colon descendens*; (11) *colon ascendens*.

The sagittal post-contrast anatomical study of *regio abdominis* in the plane 10 mm right to the median plane provided data about the retroperitoneal topographic location of the *gl. adrenalis dextra* in the *regio abdominis lateralis dextra*. The right adrenal gland appeared as an ellipse-shaped soft tissue finding in close proximity to the relatively contrast-enhancing *v. cava caudalis*. The gland was observed cranially to the right kidney. *Pars descendens duodeni* findings were found ventrally to the gland (Figure 3).

**Figure 4:**
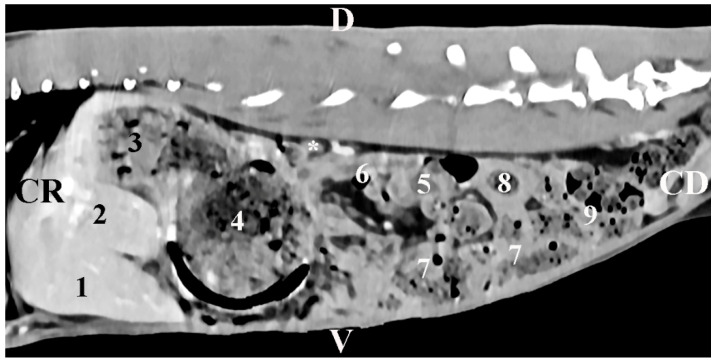
Sagittal CT post-contrast anatomical study of the rabbit abdomen at the level of the plane at 10 mm to the left of the spine. CR—cranial; CD—caudal; D—dorsal; V—ventral. (white star) mesenterial fold; (1) *lobus hepatis sinister lateralis*; (2) *lobus hepatis sinister medialis*; (3) *fundus ventriculi*; (4) *corpus ventriculi*; (5) left kidney; (6) *pars ascendens duodeni*; (7) *caecum*; (8) *colon ascendens*; (9) *jejunum*.

The sagittal post-contrast CT scan of *regio abdominis* 10 mm left to the spine showed the image of the *gl. adrenalis sinistra*. The gland was retroperitoneally located in the *regio abdominis lateralis sinistra*. The left adrenal gland was a soft tissue finding with intermediate density. The gland was observed close to the contrast-enhancing *aorta abdominalis* and the stomach. It was at a distance from the left kidney. The gland had an oval shape and distinct borders. The spinal muscles remained dorsal to the gland (Figure 4).

### 3.3. Dorsal Post-Contrast CT Anatomical Study

**Figure 5:**
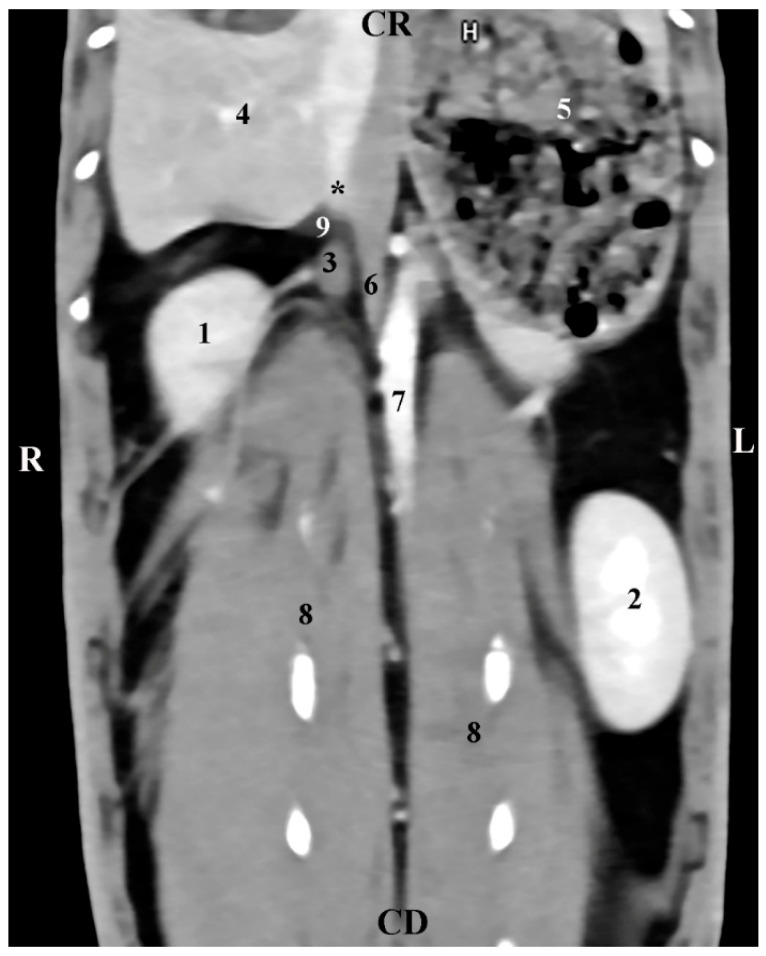
Dorsal (coronal) CT post-contrast anatomical study of the rabbit abdomen (at the plane 30 mm ventral from the vertebral column). CR—cranial; CD—caudal; R—right; L—left. (black star) right adrenal gland; (1) Right kidney; (2) left kidney; (3) right adrenal gland; (4) liver; (5) *fundus et corpus ventriculi*; (6) *v. cava caudalis*; (7) *aorta abdominalis*; (8) longissimus muscle; (9) impression of the right adrenal gland; (*) *proc. caudatus*.

The dorsal (coronal) post-contrast anatomical CT study of *regio abdominis* through the dorsal plane 30 mm ventrally to the spine showed the elliptical shape of the homogeneous and sharply delineated image of the right adrenal gland. The *gl. adrenalis dextra* was located in the *regio abdominis lateralis dextra* of the *regio abdominis* media. The gland was retroperitoneally located, medially in contact with the *v. cava caudalis,* and was observed craniomedially to the hyperattenuated borders of the right kidney and caudally to the *Proc. caudatus* of the caudate liver lobe, with which it was in anatomical contact. The border between them was defined by hypoattenuated contours of the impression of the right adrenal on the *proc. caudatus*. Dorsally, the gland was in contact with the *m. longissimus* (Figure 5).

**Figure 6:**
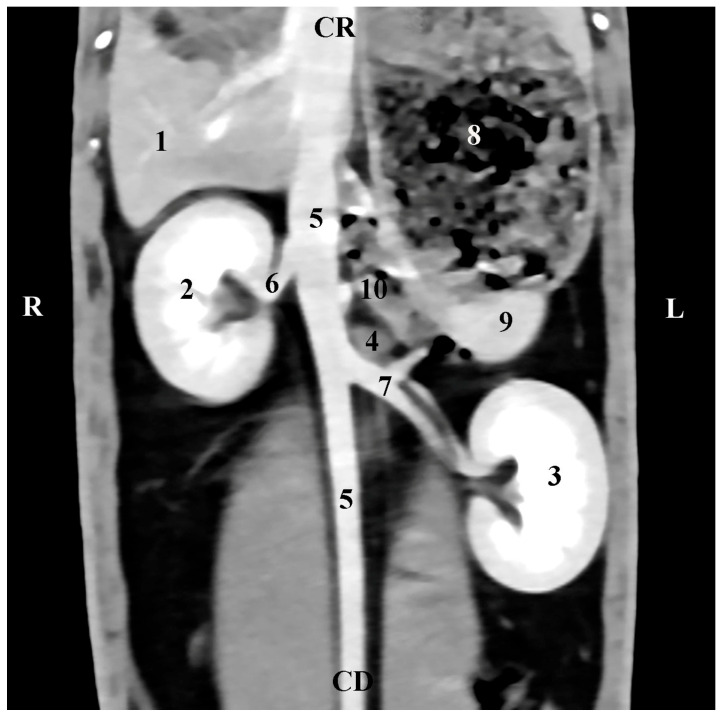
Dorsal (coronal) CT post-contrast anatomical study of the rabbit abdomen (at the plane 45 mm ventral from the vertebral column). CR—cranial; CD—caudal; R—right; L—left. (1) Liver; (2) right kidney; (3) left kidney; (4) left adrenal gland; (5) *aorta abdominalis*; (6) *a. reanlis dextra*; (7) *a. renalis sinistra*; (8) *fundus et corpus ventriculi*; (9) spleen; (10) *pars ascendens duodeni*.

The left adrenal gland was presented at the subsequent scanning level (45 mm ventrally to the spine). Its localization was in the *regio abdominis lateralis sinistra* of the *regio abdominis* media. The soft tissue features of the *gl. adrenalis sinistra* had a lower degree of attenuation in relation to the contrast-enhancing abdominal aorta, with which the gland was in direct contact medially. The contrast-enhancing *a. renalis sinistra* was observed caudally to the left adrenal gland, and the contours of the *pars ascendens duodeni* were seen cranially. The gland shape was oval. Unlike the right adrenal gland, the left gland was at a significant distance from the hyperattenuated left kidney (Figure 6).

### 3.4. CT Macrometric Results

The mean CCD of the right adrenal gland was 6.9 ± 0.5 mm and exceeded the mean CCD of the left gland, which was—5.4 ± 0.6 mm. The average value of the right DVD (5.7 ± 0.7 mm) was greater than that of the left gland, which was—4.0 ± 1.1 mm. The right adrenal LMD was lower compared to that of the left adrenal, which was—4.1 ± 0.9 mm. The values of both parameters were not sex-associated (Table 1).

## 4. Discussion

The presented CT anatomical data in the three orthogonal planes reflect the topography of rabbit adrenals in the retroperitoneal space as well as the proximities and anatomical associations of these glands with both kidneys and the large blood vessels (*aorta abdominalis* and *v. cava caudalis*). The described facts correspond to reported anatomical data [1,2] about the localization of rabbit adrenals. The more cranial location of the right adrenal gland with respect to the left gland adds to existing opinions on the mutual position of both glands [1,2].

The present established data about the cranial localization of rabbit adrenal glands vs. the ipsilateral kidney and the close anatomical right adrenal–right kidney connection, in contrast to the left adrenal gland, which is placed at a distance from the left kidney, completing the reported facts about the topography of these organs in the rabbit [1].

Our investigations and previously reported data [3,4,5,6,7,8] suggested that the presented CT anatomical data on the adrenals of the rabbit may be used as an appropriate anatomical model for the diagnostics of rabbit-specific diseases, as well as a morphological model in experimental research in other mammalian species and men.

The presented CT macromorphometric data in relation to the greater right adrenal CCD and DVD in comparison to the left adrenal gland correspond to information reported for these organs in New Zealand White rabbits [4]. In our opinion, only the MLD of the left adrenal is greater than that of the right one. Our data suggested that the values of these metric parameters are not sex-associated, confirming previously reported research data [4,19].

The greater CCD and DVD of the right adrenal gland than respective measurements of the contralateral one did not agree with data from a previous study of ours [9], which affirmed that post-mortem macrometric parameters of the left adrenal gland measured after extirpation exceeded those of the right one. Furthermore, the present data were obtained in live subjects in the field, where both adrenals were most clearly visualized. Our hypothesis is that these variations (the greater CCD and DVD parameters of the right gland, compared to the left gland, and the slightly greater values of MLD of the left gland) correlated with the body weight of the studied animals. According to us, the body weight is significant for the variations of these parameters in the rabbit.

In our opinion, the MLD of the left adrenal was greater than the right adrenal MLD. This hypothesis corresponds to information about higher left adrenal width in the porcupine [10].

The right adrenal gland’s CCD and DVM are greater than those measured for the left adrenal in both sexes. This is why the presented values support an earlier assumption about the greater length of the right adrenal gland in *Galea spixii,* yet differ from the reported greater width than the left gland’s width [11]. Our results evidence that only the right adrenal MLD in the rabbit was inferior to the same dimension of the left gland.

Macrometric measurements from the post-contrast CT study demonstrated equal tendencies in both sexes. This is not in line with the reported sex dimorphism for adrenal glands in the lowland paca [12].

Helical anatomical CT images of rabbit adrenals are of high definition and provide general information from thin CT sections. This allows supporting the existing opinion [13] for the implementation of helical post-contrast CT as a contemporary non-invasive method for obtaining objective anatomical information for a variety of soft tissue structures, including adrenal glands. The animals are studied with minimal stress to obtain anatomical data on small structures.

CT data on the anatomical relationships between the right rabbit adrenal with the right kidney, the liver, and the *v. cava caudalis*, as well as CT data on the left adrenal associations with the duodenal loops, *aorta abdominalis*, and *a. renalis sinistra*, prove our study’s contribution to the accurate interpretation of some investigations in this animal species [14]. It should be noted that our study presents objective information for the localization of both adrenal glands in relation to the spine. This approach is different from the reported study algorithm [14], which allows visualization only of the left adrenal gland on CT scans at a lower distance from the vertebral column.

The successful analysis of the CT anatomical study of the rabbit adrenal glands confirmed CT as an anatomical imaging method for the study of these organs in live animals. Therefore, our results confirm the advantages of the method in support of data published in dogs [15]. Our finding that the right adrenal CCD and DVD are greater than those of the left one, as well as the lower right adrenal MLD, differ from findings in feline adrenals (approximately equal length and height of both glands, and wider right adrenal gland) [17]. At the same time, our hypothesis adds to the described data on other animal species [18] about the greater thickness and length of the right gland, compared to the left gland.

Additionally, the selected CT scanning levels in the present study provide relevant information about the shape of adrenal glands, their topography, and their macromorphometry. The soft tissue characteristics of the glands, their shape (elliptical for the right gland and oval for the left one), as well as their proximities, complete the information about these organs in other animal species [18]. The values of the studied CT parameters are real and intravital. The postulation that the right adrenal gland CCD and DVD are greater than those of the left one agrees with the described data [18]. Only the right adrenal MLD was slightly lower than the left MLD.

Our study allows presenting the obtained results as an anatomical imaging model for investigating adrenal glands in the rabbit. Our arguments are in line with the data reported in dogs [16]. In our view, the presented results may be applied as a morphological anatomical imaging model in clinical research studies.

Our theory differs from published US data [19]. According to us, the CT parameters of the studied glands are related to the body weight, and the obtained results are not dependent on the sex or the diameter of the large vessels.

The post-contrast CT scan levels used for the anatomical study of rabbit adrenal glands provide information on the topography of glands as retroperitoneal abdominal organs.

It could be affirmed that the post-contrast helical CT study of rabbit adrenals has a number of advantages for the anatomical imaging study of these organs. This allowed us to conclude that the obtained CT results are highly informative anatomical imaging data. They may be applied in the quest for an appropriate approach for the treatment of rabbit-specific adrenal diseases.

## Figures and Tables

**Table 1:** CT anatomical dimensions of the rabbit adrenal glands (in mm).

No	Right Adrenal Gland			Left Adrenal Gland			Sex M/F
	CCD	DVD	MLD	CCD	DVD	MLD	
Rabbit 1	7.5	3.9	3.5	4.2	5.3	6	M
Rabbit 2	7.2	5.2	3.7	4.4	5.6	3.2	M
Rabbit 3	7.7	5.3	4.2	5.0	3.0	2.3	M
Rabbit 4	6.6	5.6	4.2	5.7	4.8	3.2	M
Rabbit 5	5.9	5.7	4.1	5.2	3.2	4.2	M
Rabbit 6	6.8	6.2	4.2	5.7	2.8	4.3	F
Rabbit 7	7.2	7.0	3.2	5.8	4.4	4.2	F
Rabbit 8	6.4	6.4	3.6	5.8	3.1	4.5	F
Rabbit 9	7.4	6.1	4	5.9	2.5	4.5	F
Rabbit 10	6.7	5.4	4	6.2	5.1	4.1	F
Mean ± Standard Deviation (SD)	6.9 ± 0.5	5.7 ± 0.7	3.9 ± 0.3	5.4 ± 0.6	4.0 ± 1.1	4.1 ± 0.9	

## Data Availability

The presented data in the study are available form the corresponding author. They are included in the manuscript.
